# A Novel Point-of-Care Biomarker Recognition Method: Validation by Detecting Marker for Diabetic Nephropathy

**DOI:** 10.3390/diagnostics5020177

**Published:** 2015-04-23

**Authors:** Sahana Pentyala, John Muller, Thomas Tumillo, Avijit Roy, Pooja Mysore, Srinivas Pentyala

**Affiliations:** Department of Anesthesiology, Stony Brook Medical Center, Stony Brook, New York, NY 11794-8480, USA; E-Mails: sahana.pentyala@stonybrook.edu (S.P.); jmuller018@gmail.com (J.M.); tomtumillo@gmail.com (T.T.); aviroy23@yahoo.com (A.R.); mysorp@gmail.com (P.M.)

**Keywords:** biomarker, point-of-care, antibody, diabetic nephropathy

## Abstract

Biological fluid collection to identify and analyze different disease markers is a routine and normal procedure in health care settings. Body fluids are as varied as urine, blood, mucus, cerebrospinal fluid (CSF), tears, semen, *etc.* The volumes of the collected fluids range from micro liters (e.g., tears, CSF) to tens and hundreds of milliliters (blood, urine, *etc.*). In some manifestations, a disease marker (particularly protein markers) can occur in trace amounts, yet the fluids collected are in large volumes. To identify these trace markers, cumbersome methods, expensive instruments, and trained personnel are required. We developed an easy method to rapidly capture, concentrate, and identify protein markers in large volumes of test fluids. This method involves the utilization of two antibodies recognizing two different epitopes of the protein biomarker. Antibody-1 helps to capture and concentrate the biomarker and Antibody-2 adsorbed or conjugated to nanogold beads will detect the biomarker. This method was validated in capturing and detecting lipocalin type prostaglandin-D2 synthase, a marker in urine that implicates diabetic nephropathy. A one-step collection, concentration, and detection device was designed based on this method. This device can replace many of the normal body fluid collection devices such as tubes and containers. A one-step fluid collection and biomarker capture and concentration device for rapid diagnosis of diseases has tremendous advantage in terms of cost and providing timely results.

## 1. Introduction

Biomarkers are important tools for disease detection and monitoring. They serve as hallmarks for the physiological status during the disease process [1,2]. A highly effective, clinically useful biomarker for a specific disease should be measurable in a readily accessible body fluid, such as serum, urine or saliva [3]. The search for biomarkers for early disease detection has included proteins, metabolites and other biological molecules that are altered and secreted as a consequence of the disease process, and are shed into body fluids. After collecting these body fluids, the next step is to isolate and identify the marker that will give an indication of the disease process. Unfortunately, this approach is laborious and time-consuming, as specific candidate biomarker(s) must be identified from among the thousands of intact and altered molecules in the collected body fluids.

In many disease manifestations, a marker can occur in trace amounts, yet large volumes of fluids are collected (e.g., blood and urine) [1,4,5]. It is very difficult and time consuming to process these samples to concentrate and identify specific markers for diagnosis or disease status.

Any disease or deleterious symptom in the body may result in changes in the expression of protein biomarkers [6]. Sometimes, biomarker levels can increase or decrease; other times specific markers are expressed and can be detected in body fluids, particularly in blood and urine [3]. Identifying these biomarkers can lead to determining whether a person has a disease, disorder or symptom. Body fluid collection to identify and analyze different biomarkers of diseases and symptoms became a routine procedure [7]. The volumes of the collected biological fluids range from microliters (e.g., tears, CSF) to tens and hundreds of milliliters (blood, urine, *etc.*) [8]. Identification of trace markers in large volumes of body fluids may require long times, skilled professionals and often sophisticated instruments.

This report explains the development of methods and devices to rapidly capture and concentrate protein markers contained in large volumes of test fluids, by using affinity molecules (antibodies) against specific markers adsorbed to nanogold beads. The method involves two antibodies recognizing two different epitopes of the protein biomarker. Capture antibody (AB1) is used to trap and concentrate the biomarker. Detection antibody (AB2), which was adsorbed to nanogold beads, identifies the captured biomarker. This method can replace many of the normal body fluid collection devices like tubes and containers. A one-step collection and biomarker capture and concentration device for rapid testing was also designed based on the developed methodology. This method and device will not only become an integrated sample collector and biomarker concentrator for point of care diagnosis in health care settings, but also can be utilized in clinical research to capture and concentrate trace amounts of biomarkers from large volumes of fluids.

### 1.1. Methodology Development and Design of the Biomarker Detection Kit

Generally, when a patient visits a physician’s office, body fluids are collected and sent to clinical labs for testing. The test results may come back to the physician within 24 to 48 h. Sometimes, these results may even take up to a week, depending upon the biomarker test. The device presented here is a point-of-care bedside detection kit designed to be used by a health care provider or a patient to readily identify and diagnose the disease.

The Biomarker detection kit consists of three parts.

(1) A nitrocellulose or PVDF strip that is coated with a capture antibody (AB1) that can bind to a specific biomarker in the body fluid to be tested.
(2) A tube containing a wash solution to remove other proteins non-specifically binding to the strip.
(3) A tube containing solution of gold nano beads or latex beads adsorbed with detection antibody (AB2) directed against the same biomarker protein.

### 1.2. Procedure

**Figure 1:**
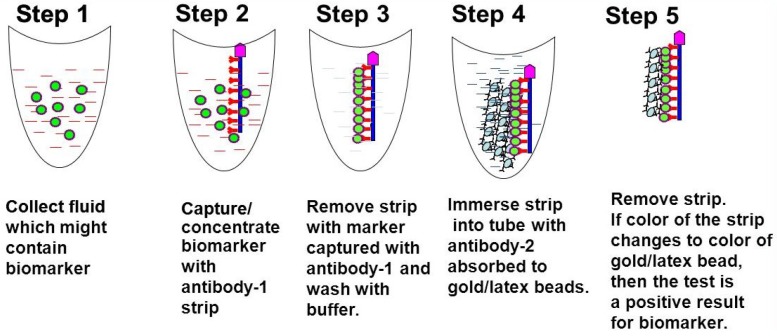
Point-of-care biomarker/disease/symptom detection kit: the kit consists of 3 parts. (1) A strip that is coated with a capture antibody (AB1) (either one side or both sides of the flat surface) that can bind to a specific biomarker in the body fluid that is tested. (2) A tube containing a solution to wash the strip so other proteins that stick to the strip nonspecifically can be removed. (3) A tube containing latex/nanogold adsorbed either/or polyclonal/monoclonal detection antibody (AB2), which is against the same biomarker protein. The test strips can be coated with multiple biomarker antibodies making this kit a multiplex detection kit where more than one disease or symptom can be identified concurrently bedside.

The strip coated with AB1 is immersed into the body fluid for 3–5 min, then removed and immersed into the wash solution tube for 3–5 min to remove nonspecific binding proteins. Then the strip is immersed into the tube containing AB2 solution. If the protein biomarker is present in the body fluid, the AB1 coated on the strip will bind to the marker and AB2 attached to gold nano beads will bind to this complex. The color of the gold will show up, indicating positivity for the disease (Figure 1).

The test strips can be coated with multiple biomarker antibodies making this kit a multiplex detection kit [9] where more than one disease or symptom can be identified concurrently at bedside. This can be achieved by adsorbing or conjugating AB2 of different biomarkers with different colored beads (gold, silver, latex, *etc.*) [10], quantum dots [11] or Surface Enhanced Raman Scattering (SERS) based nanoprobes [12].

### 1.3. Diabetic Nephropathy and Lipocalin Type Prostaglandin D2-synthase (LPGDS)

#### Validation of the Biomarker Detection Methodology by Detecting a Specific Biomarker for Diabetic Nephropathy

Urinary excretion of LPGDS was found to increase in early diabetic nephropathy [13]. Also, LPGDS is secreted in urine when there is toxicity due to high antibacterial drug use, suggesting damage to the kidneys [14]. Urinary excretions of LPGDS also reflect the increased permeability of injured glomerular capillary walls, suggesting kidney damage [15]. Urinary excretion of LPGDS is significantly increased in patients with chronic renal failure, but its diagnostic potential in less advanced stages of renal diseases remains to be elucidated [16]. Urinary LPGDS may thus predict the progression of renal injury in diabetic patients. However, there are no rapid test devices that can identify urinary LPGDS. To validate the method and the kit, an attempt was made to detect LPGDS in mock urine samples.

## 2. Materials and Methods

Nitrocellulose and PVDF membranes were purchased from Bio-Rad, Hercules, CA, USA. Gold nano beads were gifted by Nanoprobes, Yaphank, NY, USA. Mock urine was procured from Carolina Biological Supply Co., Burlington, NC, USA. LPGDS was purchased from Cayman Chemicals, Ann Arbor, MI, USA. Horse radish peroxidase (HRP) labeled goat anti mouse secondary antibody was purchased from Jackson Immunoresearch Laboratories, West Grove, PA, USA. LPGDS hybridomas were earlier produced [17,18]. All other chemicals used in the study were purchased from Sigma Chemical Co., St. Louis, MO, USA.

Along with standard lab techniques, new methods were developed and used to validate the biomarker detection device:

1. Hybridoma screening;
2. Specificity of AB1 and AB2;
3. Adsorption of capture antibody (AB1) to nanogold beads; and
4. Detection of LPGDS marker in mock urine.

## 3. Results

### 3.1. Hybridoma Screening

**Figure 2:**
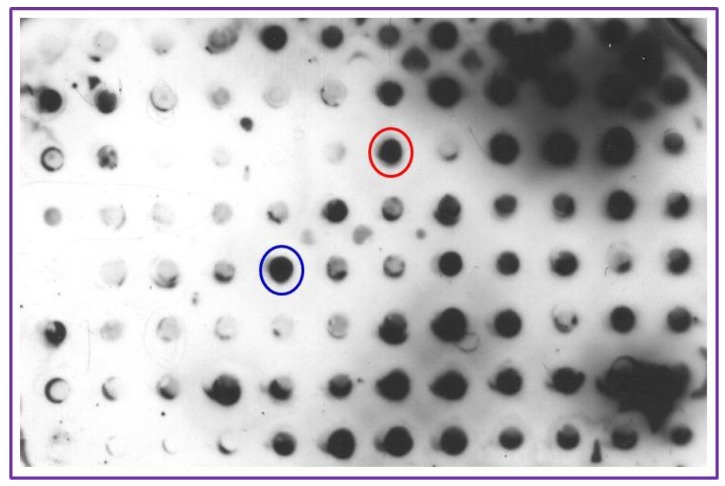
Hybridoma screening for capture and detection antibody selection: LPGDS (pure protein 20 ng) was transferred to PVDF membrane using a Bio-Rad dot blot apparatus and probed with ascitic fluid generated from culturing different hybridoma clones against LPGDS. The membrane was developed using ECL detection kit. Dark dots indicate positive interaction. Positive antibody from red circle was designated as Antibody 1 (AB1) and that in the blue circle is designated as Antibody 2 (AB2) for use in the biomarker detection method. Other clones showed specificity at different levels and these can also be used to detect LPGDS. AB1 and AB2 were selected based on their strong interaction with LPGDS.

To select the capture (AB1) and detection (AB2) antibodies from clones earlier generated, hybridoma clonal screening was performed. PVDF membrane was cut to grid size of the Slot Blot apparatus (BioRad, Hercules, CA, USA) and presoaked in methanol and washed with water and assembled onto the apparatus. All the reagents were added using narrow-mouthed pipet tips to individual grid slots without dismantling the apparatus. Draining of the fluids from the assembled grid was achieved by connecting the apparatus to a vacuum pump and switching on the vacuum when needed. LPGDS (10 ng in 20 µL) was deposited into the center of each grid slot. The membrane was allowed to dry for 1 h blocked in 5% milk (200 µL) for 1 h and washed with Tris buffered saline (TBS, 10 mM Tris-HCl, pH 7.5, 150 mM NaCl,) three times for 5 min each. Ascetic fluid (200 µL) from different clones was dispensed separately into individual grid slots and incubated for 1 h, drained and washed with TBS three times. The apparatus was dismantled and the PVDF membrane was lifted and incubated with horseradish peroxidase conjugated 2nd antibody in a solution of 3% milk/TBS for 1 h. After washing the membrane in TBS, positivity of different clones was visualized using enhanced chemiluminescence reagent (GE Healthcare, Pittsburgh, PA, USA) and the images were captured using a C-digit blot scanner (LICOR, Inc., Lincoln, NE, USA). Two clones with strong reactivity were identified (circled spots on Figure 2) and their corresponding antibodies were designated as AB1 (capture antibody) and AB2 (detection antibody).

### 3.2. Specificity of AB1 and AB2

**Figure 3:**
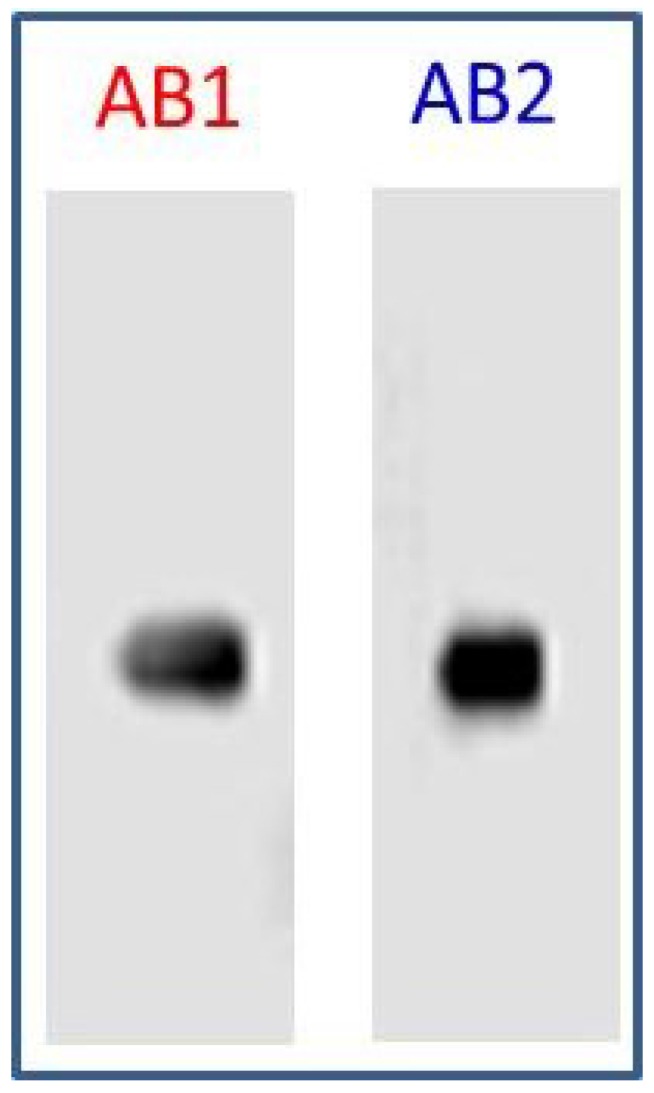
Specificity of capture (AB1) and detection (AB2) antibodies: To validate the utility and specificity of the selected antibodies (AB1 and AB2), LPGDS (20 ng) was resolved on a 12% SDS-PAGE, blotted to PVDF membrane, blocked with 3% milk in TBS and probed with AB1 and AB2 culture supernatants. Secondary antibody is HRP labeled goat anti mouse from Jackson Laboratories (1:5000) and HRP labeled goat anti rabbit from Biorad (1:300). Protein bands were detected by ECL (GE Healthcare) with a LICOR C-digit reader (scan time 6 min).

To confirm the specificity of AB1 and AB2 for its ability to bind to LPGDS with strong affinity, immunodetection methods were used. LPGDS (10 ng) was subjected to electrophoresis on 12% polyacrylamide gels. After loading the gel with LPGDS, electrophoresis was performed for 1.5 h at 90 volts using a Mini-Protean II PAGE system (Bio-Rad). Resolved proteins on the gels were electroblotted to Immobilon PVDF membrane (Bio-Rad) using Transblot Semi Dry Transfer apparatus (BioRad) at 17 V for 1 h with transfer buffer consisting of 25 mM Tris-base, 192 mM glycine and 20% methanol. After transfer, the membrane was blocked with non-fat milk (3%) suspended in TBS for 1 h, and then incubated with AB1 or AB2 solutions at room temperature for 1 h. Membranes were washed with TBS and then incubated with horseradish peroxidase conjugated 2nd antibody in a solution of 3% milk/TBS for 1 h. The LPGDS protein was visualized (Figure 3) by processing the membrane with enhanced chemiluminescence reagents (GE Healthcare) and the images are captured using a C-digit blot scanner (LICOR).

### 3.3. Adsorption of Detection Antibody (AB2) to Nanogold Beads

**Figure 4:**
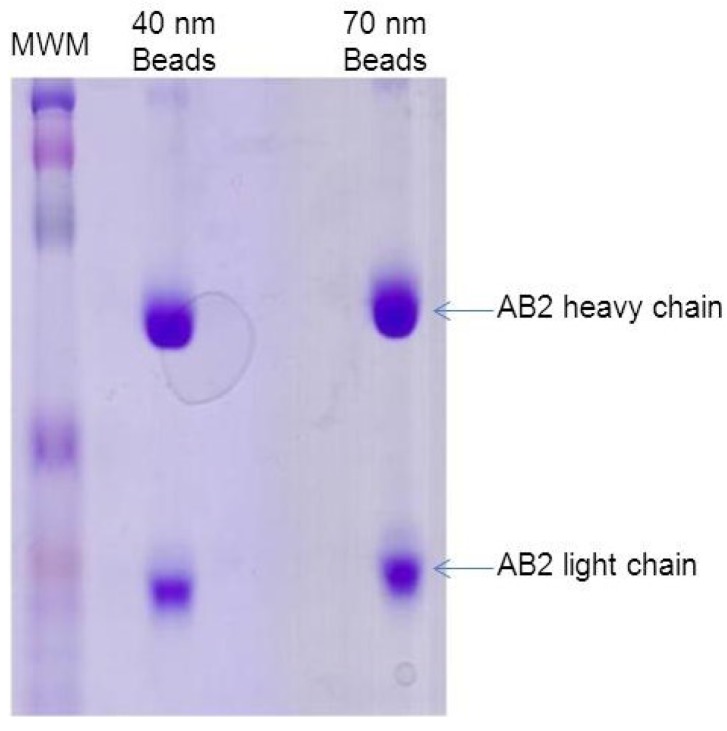
Adsorption of AB2 to gold nano beads: 40 and 70 nm gold beads were incubated with AB2 and resolved by SDS-PAGE. (MWM = Molecular weight marker).

Gold nano-beads (40 and 70 nm diameter) were obtained from Nanoprobes (Medford, NY, USA). Adsorption of AB2 (LPGDS detection antibody) to 40 and 70 nM gold beads was tested to identify the appropriate gold bead size for the studies. 10 mL of 40 nM gold bead suspension was spun down at 1700 rpm. The supernatant was removed and the beads were washed with 5 mL of phosphate buffered saline (PBS) and placed in the centrifuge. This step was then repeated. Finally, 6 mL of AB2 was added to the beads and incubated at 4 °C over night with gentle rocking. The AB2-gold bead solution was prepared by centrifuging the solution at low speed (~950 rpm) for 5 min. The supernatant was decanted and the pellet material washed with 2 mL of PBS and spun at a low speed for 5 min. Gold bead attached AB2 was re-suspended in 1 mL of PBS and transferred to a test tube for further use. Binding of the antibodies to the gold nano-beads was confirmed by SDS-PAGE. Resolution of the two bands (light and heavy chains of the antibody) confirmed the adsorption of AB2 to gold nano beads (Figure 4). Results showed that both 40 and 70 nm beads had similar affinity for AB2. Gold beads of 40 nM were selected for the experiment as the relatively small size of the particles renders them more mobile for interaction in liquid media.

### 3.4. Detection of LPGDS (Diabetic Nephropathy Marker) in Mock Urine

#### 3.4.1. Preparation of Test Strips with LPGDS Antibody

PVDF membrane was cut to appropriate size to fit the Slot-Blot apparatus. The membrane was soaked in methanol for one minute to make it hydrophilic and then washed in distilled water twice for 2 min each. The membrane was placed in the slot-dot apparatus and connected to a vacuum. Once a tight seal was formed to trap the membrane on the apparatus (tested with a distilled water drop), 200 μL of AB1 (LPGDS capture antibody) was added in designated rows. Row E was selected and AB1 was added to slots of columns 1 through 4. PBS was added to slots in column 5 and 6 instead of AB1, which served as control. After addition of AB1 to designated slots, the membrane was left to dry for 1 h.

#### 3.4.2. Detection of Disease Marker (LPGDS) in Urine Samples

**Figure 5:**
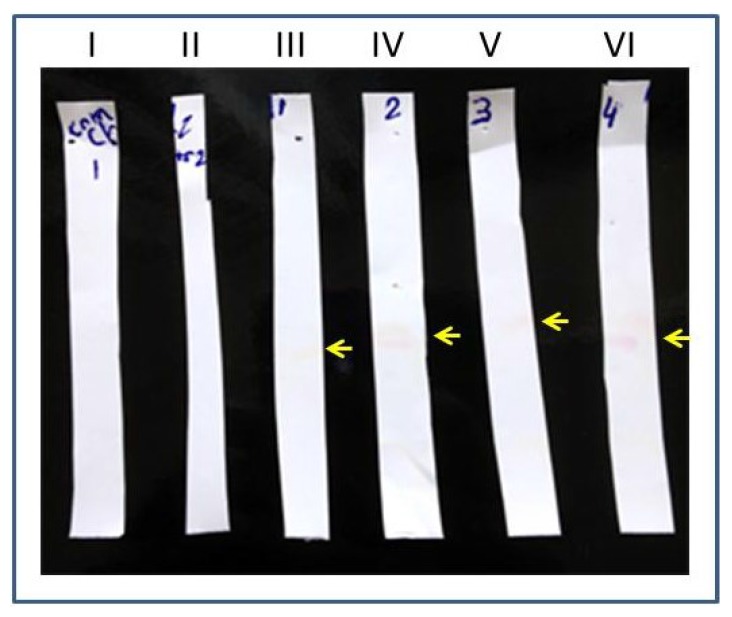
Detection of diabetic nephropathy marker (LPGDS): AB1 coated strips are incubated in LPGDS spiked mock urine samples. AB2 coated nanogold beads were allowed to interact with the strips. Marker detection (pointed by yellow arrows) was visualized by pink colored band (gold beads color). Strips I and II are control and strips III to VI are experimental.

**Figure 6:**
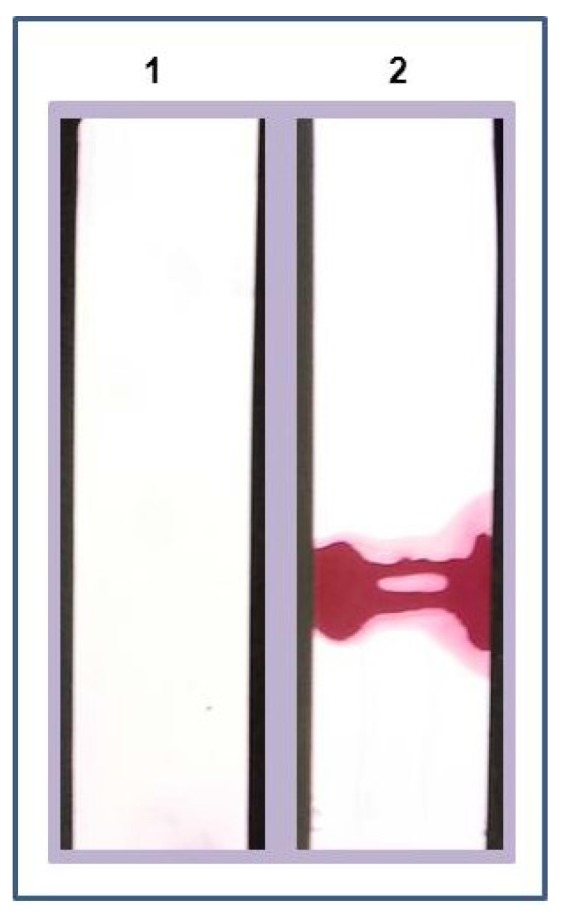
Enhanced detection of marker: AB1 coated strips are incubated in LPGDS (marker) spiked mock urine sample. AB2 coated nanogold beads prestained with Ponceau-S dye were allowed to interact with the strips, washed with water, and dried. Marker detection was confirmed by dark red band on the strip (Strip1 = Control (without marker); Strip 2= experimental (with marker)).

The AB1 adsorbed membrane was cut into strips and immersed into test tubes with 2 mL of mock urine spiked with LPGDS (100 ng). After 1 h, the strips were removed from the test tubes and were washed twice in TBS for 5 min each. Strips were air dried for 10 min and each strip was immersed into a test tube containing AB2 (detection antibody adsorbed to gold beads) for 10 min. The strips were pulled out of AB2 solution and air dried for 30 min. Experimental strips coated with AB1 showed positivity (pale pink color bands) as AB2 (detection antibody) adsorbed to the gold beads interacted with the captured marker protein (strips III to VI in Figure 5). Control strips (that were not coated with AB1-capture antibody) did not show any bands (strips I and II in Figure 5). To more clearly visualize the interaction of the marker and the AB2 adsorbed to gold beads, Ponceau-S detection method was used (Ponceau-S is a diazo sodium salt negative stain with light red color that non-covalently binds to the positively charged amino groups of non-polar regions in the protein and does not interfere with antibody-antigen binding). The experiment was performed using control and experimental strips (nitrocellulose). The only modification is that AB2 adsorbed to gold nano beads was pre stained with Ponceau-S dye. The AB2-bead complex was incubated with 1 mL of the dye for 10 min, washed and re-suspended in 1 mL PBS before using in the detection step of the experiment. The detection of the LPGDS marker is strongly visible on the experimental strip by the appearance of dark red color band (Figure 6).

## 4. Discussion

The need for a rapid, reproducible, sensitive and simple diagnostic test is of major importance in healthcare. Such a test has the obvious advantage over the existing laboratory tests, *i.e.*, radio-immuno assay [19], immunofixation electrophoresis [20], enzyme-linked immunosorbent assay (ELISA) [2] and immunoblotting [21], *etc.*, in that it can be performed immediately beside the patient giving a result in a few minutes of time instead of several days when the sample is sent for analysis to a laboratory. Many of the existing laboratory tests are time consuming and need trained personnel and expensive equipment [22]. A novel immunochromatographic test, based on the use of biomarker specific antibodies was designed and this method was validated by detecting LPGDS, a biomarker for diabetic nephropathy. The results show that this novel detection method can identify unique biomarkers that are expressed in response to a disease or a symptom. This novel method is easy to use and can readily be used at the bedside as a point-of-care diagnostic with a step-by-step easy to use protocol even by untrained personnel. This method at its present stage cannot quantify the biomarker concentration (up or down-regulation of biomarkers which can give diagnosis or prognosis). Further modifications are possible to quantify the biomarkers by labeling antibodies with different fluorescent tags and analyzing with fluorescent readers. Efforts are under way to test LPGDS detection method and kit with urine samples procured from control and diabetic subjects who are diagnosed with nephropathy at Stony Brook University Hospital, and pending approval for human subjects study. Validation with clinical samples will make this invention the first ever method and kit to rapidly detect diabetic nephropathy.

## 5. Conclusions

The integrated fluid collector and biomarker concentrator will be able to capture trace biomarkers and provide prognosis and diagnosis of a disease or symptom right at the collection site. This project will result in the development of a rapid point of care detection kit for diseases like diabetic nephropathy. The use of the proposed method and kit has several advantages against other collection and detection methods. This is a simple method and the test procedure is very rapid. Sample preparation and test performance require no extra equipment. The kit can be used in any point of care situation by the health care industry. Thus, the test can easily be performed in small clinics as well as by patients themselves to capture biomarkers without complex lab equipment. This kit can replace many of the normal collection devices like blood, plasma, and urine collection tubes and containers. The kit will be able to identify, capture and concentrate the specific marker that is to be tested in a rapid fashion bedside. The methods and devices that were proposed in this project can even be used to detect substances of abuse. As such, this innovative device and detection methods will be of immense value to the health care and diagnostics industry.

The necessity for an ideal fluid collection and biomarker concentrating and detection device is important in the diagnostics field. There is a potential application of this integrated collection and capture kit in many health related scenarios (any disease or symptom that utilizes body fluids for diagnosis). New technologies like detecting biomarkers with digital PCR [23], electronic nose technologies for volatile markers [24], nanostructures based electrosensors [25] and smart phones encompassing conventional colorimetric detection [26,27] are already being developed in the field of diagnostics. Further development of these technologies will have an impact on how quickly a disease can be diagnosed. Our novel method can be developed into a multiplex detection as well as a biomarker quantification kit which will have immense value as it can diagnose and prognose multiple diseases in one-step. Low cost and ease of use will be of immense value for not only developed countries where health care costs are large, but will also be of great help in poor and developing countries.
